# Dietetic Notes

**Published:** 1908-05-16

**Authors:** 


### DIETETIC NOTES.

### PERCENTAGES OF IRON IN FOODS.

The following analyses of various foodstuffs for iron may be of interest in connection with the dietaries of persons suffering from chronic anæmia, to whom it is desired to give iron in the form of natural foods rather than as medicine. The figures are for the dried substances, so that, in estimating the total amounts of iron that the patient may be receiving in the food in each twenty-four hours, account must be taken of the percentage of water that the various foods contain. The figures represent the number of milligrams of iron contained in 100 grams of the dry substances.

| | | | |
|---|---|---|---|
| White bread ... ... | 1.4 | Potatoes ... ... | 6.2 |
| Apples, sweet ... | 1.7 | Green peas ... ... | 6.8 |
| Apples, sour ... ... | 2.1 | French beans ... | 8.5 |
| Pears ... ... ... | 2.2 | Carrots ... ... | 8.9 |
| Cow's milk ... ... | 2.3 | Lentils ... ... | 9.3 |
| Goat's milk ... ... | 2.5 | Asparagus ... ... | 10.5 |
| Brown bread ... ... | 2.5 | Yolk of egg ... ... | 18.3 |
| Red currants ... ... | 3.6 | Green chicory ... | 22.0 |
| Rice ... ... ... | 4.5 | Cabbage ... ... | 30.5 |
| Barley ... ... | 4.7 | Spinach ... ... | 40.0 |
| Black grapes ... ... | 5.8 | | |

The relatively large amount of iron in spinach and cabbage may partly account for the old maxim that "green vegetables are excellent for the blood."

